# Effects of the Structure of TiO_2_ Nanotube Arrays on Its Catalytic Activity for Microbial Fuel Cell

**DOI:** 10.1002/gch2.201800084

**Published:** 2018-10-25

**Authors:** Tao Guo, Changzheng Wang, Ping Xu, Cuimin Feng, Shuai Si, Yajun Zhang, Qiang Wang, Mengtong Shi, Fengnan Yang, Jingxiao Wang, Yang Zhang

**Affiliations:** ^1^ Key Laboratory of Urban Stormwater System and Water Environment Ministry of Education Beijing University of Civil Engineering and Architecture Beijing 100044 P. R. China; ^2^ Laboratory for Micro‐sized Functional Materials College of Elementary Education Capital Normal University Beijing 100048 P. R. China; ^3^ Beijing Institute of Nanoenergy and Nanosystems Chinese Academy of Sciences Beijing 100083 P. R. China

**Keywords:** COD, microbial fuel cells, photocatalytic, power generation, TiO_2_ nanotube arrays

## Abstract

To enhance the microbial fuel cell (MFC) for wastewater treatment and chemical oxygen demand degradation, TiO_2_ nanotubes arrays (TNA) are successfully synthesized on Ti foil substrate by the anodization process in HF and NH_4_F solution, respectively (hereafter, denoted as TNA‐HF and TNA‐NF). The differences between the two kinds of TNA are revealed based on their morphologies and spectroscopic characterizations. It should be highlighted that 3D TNA‐NF with an appropriate dimension can make a positive contribution to the high photocatalytic activity. In comparison with the TNA‐HF, the 3D TNA‐NF sample exhibits a significant enhancement in current generation as the MFC anode. In particular, the TNA‐NF performs nearly 1.23 times higher than the TNA‐HF, and near twofold higher than the carbon felt. It is found that the two kinds of TiO_2_‐based anodes have different conductivities and corrosion potentials, which are responsible for the difference in their current generation performances. Based on the experimental results, excellent stability, reliability, and low cost, TNA‐NF can be considered a promising and scalable MFC bioanode material.

## 1 Introduction

Wastewater treatment is one of the most important concerns in the modern society and sanitation needs in protecting the water bodies that served as the sources of drinking water.^1^, ^2^ There are plenty of treatment technologies for domestic wastewater. Nowadays, the prevailing activated sludge consumes 0.6 kW m^−3^ to treat the chemical oxygen demand (COD) in domestic wastewater.^3^ It is ≈4% of the electricity consumption in the United States for wastewater treatment, which is about 110 TWh year^−1^, or equivalent to 9.6 million households' annual electricity consumption.^4^ Within the United Kingdom, wastewater treatment requires almost 6.34 GWh of electricity, ≈1% of the average daily electricity consumption of England and Wales.^5^ Previous works indicated that there is an abundance of energy in wastewater. For instance, chemical energy (≈26%) is available in the forms of carbon by measuring COD and nutrient compounds (e.g., nitrogen and phosphorous), which urge us to exploit new energy harvesting and environment‐friendly treatment technologies.^6^, ^7^, ^8^

Direct disposal of wastewater generating from various sources such as industrial, domestic, and agricultural facilities is mainly responsible for environmental impacts including eutrophication of surface waters, hypoxia, and thermal pollution impairing potential drinking water source.^6^, ^9^, ^10^ In particular, the wastewater from pharmaceutical factories, breweries, landfills, and food processors has various organic compounds.^11^, ^12^ Microbial fuel cell (MFC) as a green technology to turn organic wastes into electrical energy has attracted considerable attentions in the past decade, which can be considered a potential solution to solve the environmental and energy problems.^13^, ^14^ However, it is found that the rapid development of MFC technology has been largely limited by the low power generation efficiency which includes the high cost of electrode materials and the insufficient charge transfer between microorganisms and electrodes.^15^, ^16^ Great efforts have been devoted to seeking suitable materials and their modifications to deal with these issues, including carbon/graphite materials, carbon nanotubes, conducting polymers, and metal nanoparticles.^17^, ^18^, ^19^, ^20^, ^21^, ^22^, ^23^, ^24^, ^25^, ^26^ Nevertheless, their applications are still restricted in terms of low specific surface area, poor biocompatibility, low corrosion resistance, and/or complicated synthetic procedures.^18^, ^27^ Therefore, the rational design and synthesis of highly efficient and stable electrode materials have an essential impact on the MFC applications.

The efficiency of a MFC electrode can be improved by using low‐cost materials without significantly sacrificing their performances.^28^, ^29^, ^30^ As a key part of bioelectrochemical system, electrode materials play a vital role in boosting MFC performance and reducing the cost. Because of the excellent corrosion resistance, mechanical property, and conductivity, titanium foil has been widely investigated as a multidimensionally stable anode in the electrolysis industry.^31^, ^32^ Titanium dioxide (TiO_2_) served as one of the most attractive metal oxides that has received tremendous attentions due to its biocompatible and stable characteristics.^33^ Thanks to its abundance, nontoxicity, and chemical stability, TiO_2_‐based materials have great potential in photocatalysis, cosmetic, paints, lithium‐ion batteries, and dye‐sensitized solar cells.^34^, ^35^, ^36^, ^37^, ^38^, ^39^, ^40^ TiO_2_ nanoparticles have been employed to modify carbon electrode to increase the power output of the MFC.^23^, ^41^, ^42^, ^43^ TiO_2_ nanotubes with large surface area have been widely studied for their photovoltaic and photocatalytic applications.^44^, ^45^, ^46^, ^47^ In these regards, we reported the fabrication of an anode of MFC based on the TiO_2_ nanotubes arrays (TNA). The sludge supernatant from the wastewater treatment plant was used as the main source of microorganism. The morphologies, crystal structures, electrochemical activities of as‐fabricated electrodes and their power output of the MFCs were systematically investigated. Our results indicated the maximum power density of the MFC loading with 3D TNA as anode has been markedly improved which paves the way for the development of MFC technology.

## 2 Results and Discussion

### 2.1 Synthesis and Characterization of TNA‐Based Electrodes

**Figure 1:**
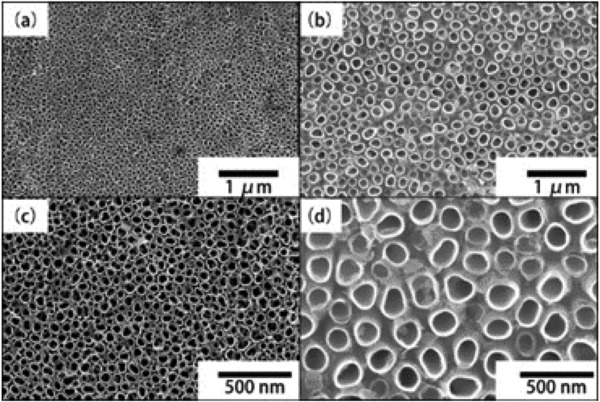
a,c) SEM image of top view of TNA‐HF. b,d) SEM image of top view of TNA‐NF.

Two‐step anodization was adopted to better control the surface morphology in the synthetic process of TNAs. In the first anodization step, the bowl‐like footprints were left on the titanium substrate. The second step of anodization process, Ti is dissolved and accumulated nanopores. At the same time, the dissolution of TiO_2_/Ti(OH)_4_ in the presence of fluoride is the key in controlling the surface morphology.^48^, ^49^, ^50^ The morphology of as‐synthesized TiO_2_‐based nanotubes arrays was investigated by scanning electron microscopy (SEM) characterization. **Figure** **1** shows the SEM images of TNA‐HF and TNA‐NF obtained in HF and NH_4_F solution, respectively. The digital image of Ti foil reveals the color of Ti foil changes from silver to yellow/blue, indicating that TNA was formed on the Ti foil surface (see Figure S1, Supporting Information). TNA on Ti foil were extended in 3D morphology, which may possess much more excellent catalytic active sites. As shown in Figure 1c, the TNA‐HF have an average diameter ranging from 50 to 120 nm. The thickness of TNA‐HF is between 10 and 25 nm and tube pitch of TNA‐HF increased from 5 to 50 nm with a pore density of ≈7.5 × 10^10^ cm^−2^ in comparison with TNA‐HF. It is found that the TNA‐NF has larger diameter of nanotubes ranging from 80 to 150 nm. The average thickness of TNA‐NF is thinner (5–10 nm), and the tube pitch is larger (50–150 nm) than TNA‐HF (see Figure 1d). TNA‐NF are interspaced by several gaps, with a pore density of ≈2.0 × 10^10^ cm^−2^, which can be attributed to van der Waals attraction and capillary force during drying.^2^, ^51^ As shown in Figure S2 (Supporting Information), the nanotubes diameter distribution and density of nanotubes of the two kinds of TNA are clearly different. The differences in TiO_2_ nanotube diameter may have a significant impact on the biofilm growth and tune number of active sites for microorganism.^52^, ^53^, ^54^

Previous reports indicated the process of chemical dissolution is related to the concentration of F ion in NH_4_F and HF solution. The formation of TNA can be explained by the following equations^55^, ^56^, ^57^, ^58^

(1) $$\mathrm{Ti}-4e^{-}$$

(2) $${\mathrm{Ti}}^{4+}+2H_{2}O\to {\mathrm{TiO}}_{2}+4H^{+}$$

(3) $$\left(\mathrm{or}:{\mathrm{Ti}}^{4+}+\;\;4H_{2}O\to \mathrm{Ti}{\left(\mathrm{OH}\right)}_{4}+4H^{+},\mathrm{Ti}{\left(\mathrm{OH}\right)}_{4}\to {\mathrm{TiO}}_{2}+2H_{2}O\right)$$

**Figure 2:**
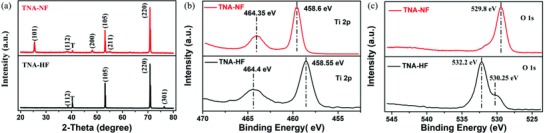
a) XRD patterns of the TNA‐HF and TNA‐NF (T‐Ti foil). XPS spectra of a) Ti region and b) O region.

The X‐ray diffraction (XRD) patterns of TNA‐HF and TNA‐NF are shown in **Figure** **2**a. The diffraction peaks can be identified at 2θ = 25.3°, 38.6°, 48.1°, 53.9°, 55.1°, and 70.3° corresponding to the (101), (112), (200), (105), (211), and (220) crystalline planes of TiO_2_. According to the XRD results, anatase is the dominant phase in both the TNA‐HF and TNA‐NF samples. As calcination was performed at 500 °C, no rutile phase was observed.^33^, ^59^ Figure 2b,c shows the high‐resolution X‐ray photoelectron spectroscopy (XPS) spectra of the Ti and O regions, respectively. The TNA‐NF exhibited two characteristic peaks (Ti 2p_3/2_ and Ti 2p_1/2_) at binding energies of 458.6 and 464.35 eV, which confirmed the existence of Ti^4+^. Similarly, TNA‐HF sample exhibited two signals at 458.55 and 464.4 eV. The broadness of Ti peaks indicated the multiple oxidation states on the TNA surface.^60^ According to previous reports, Ti^4+^ may capture photogenerated electrons and form Ti^3+^ in TiO_2_‐based materials, which can in principle promote the separation of photogenerated electrons and holes. In addition, the XPS spectra of O 1s are exhibited in Figure 2c. Previous publications reported the binding energies of lattice oxygen and adsorption oxygen are around 528.5–529.7 and 530.54–533.77 eV, respectively. The TNA‐NF exhibited a clear typical Ti—O=O bond at 529.8 eV while the dominated peaks at 532.2 eV (Ti—O bond) were unambiguously observed on the TNA‐HF surface. Although both the electrodes have lattice oxygen and adsorption oxygen, TNA‐NF has more lattice oxygen on the TNA surface. Therefore, it will be interesting to investigate whether this difference can be developed to rationally design the anode materials and further improve the MFC performance.

The electron transfer process is achieved through the physical contact of the bacterial outer membrane cytochrome C protein (OMCs), the cilia, and the anode, or other indirect pathways.^61^, ^62^, ^63^ As the previous work introduced, the surface group of TiO_2_, such as hydroxyl, can enhance the attachment of bacteria. The nanostructure of TiO_2_ contributes to the direct transfer of redox proteins to TiO_2_ as well.^64^ In addition, the electrode with nanotube arrays architecture has an additional significant advantage in promoting the movement of electrons from OMCs to electrodes, which is somehow similar to the case of photoexcited electrons effectively transfer along the orientation of TiO_2_ nanotube.^65^

### 2.2 Electrochemical Characterization of TNA Electrodes

**Figure 3:**
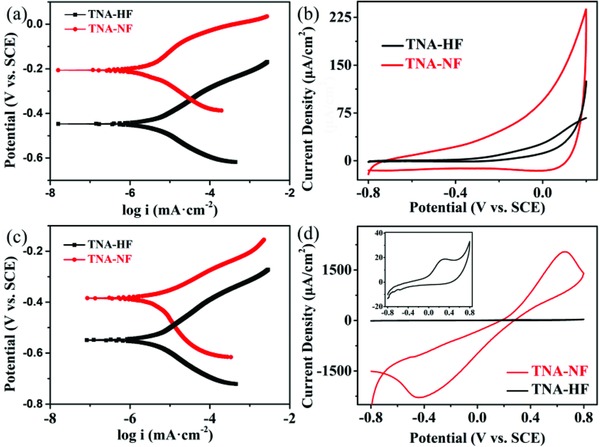
Tafel plots of the TNA‐HF and TNA‐NF electrodes: a) before and c) after the experiment. Cyclic voltammetry curves of the TNA‐HF and TNA‐NF electrodes: b) in PBS buffer solution and d) in 0.1 m K_3_[Fe(CN)_6_] and 0.1 m KCl solution, inset shows the enlarged curve of TNA‐HF.

All electrochemical measurements were performed in a three‐electrode cell. The Tafel plots in this work are quoted relative to a saturated calomel electrode (SCE). The corrosion potentials of as‐fabricated anodes in PBS were compared in the aspect of their Tafel plots. **Figure** **3**a,c shows the TNA‐HF corrosion potential is more negative than TNA‐NF which would hinder its development as stable bioanode. The 3D TNA‐NF with an appropriate dimension may provide more suitable space and/or site for the survival of microorganisms. Although the corrosion potential of TNA‐HF electrode had less change, it can be explained by its narrow space which actually has less contribution to the electron transfer process. Based on the abovementioned characterizations, TNA‐NF sample demonstrated the prospect of TiO_2_‐based bioanode.

Cyclic voltammograms (CVs) of TNA‐HF and TNA‐NF in PBS buffer are presented in Figure 3b. The area of the CVs indicated the relatively high surface area of TNA‐NF compared with the TNA‐HF. The most rectangular shape of TNA‐NF curves indicated its good conductivity. As revealed from Figure 3d, the characteristic curves pointed out that irreversible reactions took place at the TNA‐NF and TNA‐HF electrodes. The TNA‐NF and TNA‐HF electrodes were further characterized in 0.1 m K_3_[Fe(CN)_6_] and 0.1 m KCl solution, respectively. It can be concluded that the TNA‐NF had better electronic transfer ability in comparison with TNA‐HF.

On the basis of electrochemical impedance analysis, the surface capacitance of the TNA‐based electrodes increased from 0.07767 mF cm^−2^ (TNA‐HF) to 0.1769 mF cm^−2^ (TNA‐NF), which pointed out that the effective contact areas with the microorganisms of TNA‐NF were almost three times larger than that of TNA‐HF. In addition, the impedance of TNA‐HF was found to be ≈7500 Ω, which was fivefold higher than TNA‐NF (≈1500 Ω) (see Figure S3, Supporting Information), indicating that the lower impedance taking account of improving the performance of TNA‐based electrodes.

### 2.3 Evaluation of Electrode Performance

**Figure 4:**
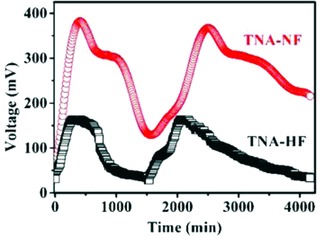
Voltage output over time of MFC with TNA‐HF and TNA‐NF as electrodes.

The power output performance of TNA electrodes was measured in a dual‐chamber reactor. **Figure** **4** shows the voltage generation for TNA‐HF and TNA‐NF electrodes. The maximal voltage of TNA‐NF was nearly 400 mV, which is much better than TNA‐HF and carbon felt. The maximal power density of TNA‐NF was 157 mW m^−2^ (see Figure S4, Supporting Information), which was nearly 1.23 times higher than that of TNA‐HF (127 mW m^−2^). In the previous work, the maximum power density of the active carbon fiber felt was 33.5 ± 1.5 mW m^−2^, and the maximum power density of graphite felt increased to 74.5 ± 7.5 mW m^−2^, which is not comparable to the TNA‐based electrode in this work.^66^ Due to the differences in their morphologies/dimensions and spectroscopic characterizations, the TNA‐NF and TNA‐HF revealed the different production capacity. Nonetheless, both of them demonstrated better performances than the carbon felt.^67^ The TNA‐based electrodes with enhanced surface area made better use of anolyte. It should be noted that the nanotubes with larger diameter and interspace have improved performance in charge transfer and material transfer, which is consistent with our aforementioned electrochemical impedance spectroscopy (EIS) results. The 3D nanotubes arrays architecture may provide more available surface for microorganisms to survive, which probably improved the power density output for TNA‐NF.

**Figure 5:**
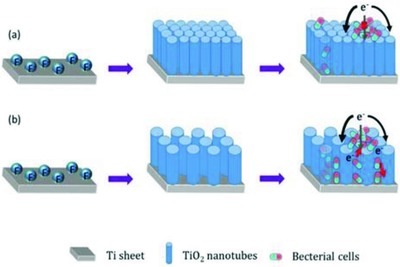
Schematic diagram of the structure of the microorganism on two electrodes: a) TNA‐HF and b) TNA‐NF.

Due to the significant differences in the dimension of TNA (Figure S2, Supporting Information), a possible mechanism is proposed in **Figure** **5**. The TNA‐NF has more interspace so that the microorganism could survive not only on the top of TiO_2_ nanotube arrays but among nanotubes as well. On the macro level, the TNA‐NF had larger nanotube diameter than TNA‐HF, which may provide more suitable room for microorganism to grow up which indeed have an essential influence on the power density generation. The electrons transport only goes along the adjacent nonconductive bacteria cells, leading to the TNA‐HF having poor power density. However, the TNA‐NF may trap the bacteria cells into the nanotubes or interspace between nanotubes and reduce electron transfer between the nonconductive bacteria cells and conductive Ti substrate, which resulted in the improvement of the MFC performance.^68^

**Figure 6:**
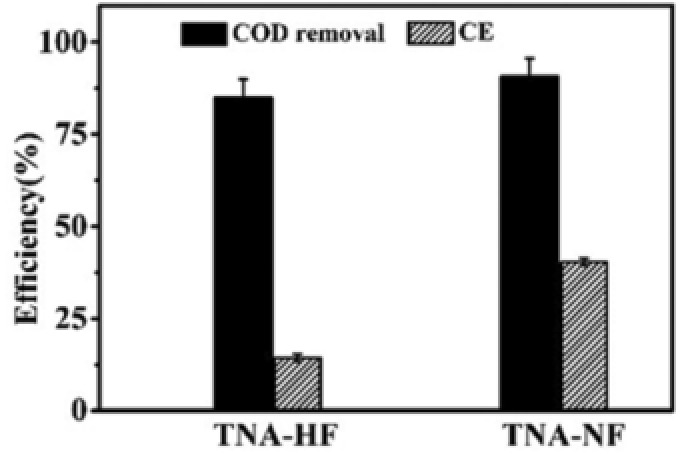
COD and coulombic efficiency (CE) for MFC with TNA‐HF and TNA‐NF as electrodes.

The COD of anode chamber was tested by ultraviolet–visible spectrophotometer (DR6000, Hitachi), which was the nutrition of microorganism. As shown in **Figure** **6**, the degradation of COD and CE (Coulombic efficiency) for TNA‐HF and TNA‐NF were different. Degradation activity of methylene blue (MB) in the presence of TNA‐HF and TNA‐NF under visible light irradiation is illustrated in Figure S5 (Supporting Information). Moreover, CE indicates the efficiency of microorganism degradation of organic pollutants into electrical energy. Because the TiO_2_ nanotubes arrays were synthesized on Ti foil, the electron transfer ability is enhanced. TNA‐NF which had larger nanotube diameters took the advantage of charge transfer and material transfer rate.^69^ With the consideration of the species diversity of microorganism, not all the microorganisms can generate electric power. However, all the microorganisms have lived up with nutrition (COD), which is the reason that two electrodes had different CE

(4) $$\mathrm{Degradation}\;\mathrm{efficiency}\;\left(\%\right)=\frac{C_{0}-C}{C_{0}}\;\;\times \;\;100\%$$

*C*_0_: the initial concentration of COD

*C*: the concentration of COD during reaction

(5) $${\mathrm{CE}}_{C\;O\;D}=\frac{8\int_{0}^{t_{b}}Idt}{FV_{\mathrm{An}}\Delta \;\;\mathrm{COD}}$$

*I*: the corresponding current at time *t* (mA)

*t*_b_: periodic time (min)

*F*: Faraday's constant (96 485 C mol^−1^)

*V*_An_: the total volume of the anodic liquid (L)

ΔCOD: change of COD concentration (mg L^−1^)

## 3 Conclusions

In summary, we successfully fabricated two types of TiO_2_‐based nanotube arrays on Ti foil by anodization in different chemical environments. In comparison with the TNA‐HF, the TNA‐NF demonstrated higher current generation in the MFC application. The maximal current density of TNA‐NF was 157 mW m^−2^, which was nearly 1.23 times higher than that of TNA‐HF (127 mW m^−2^). Our results highlighted the importance of rational design of the optimum dimension to achieve higher current generation, which is essential for practical application of MFC technology. In contrast to TNA‐HF, the TNA‐NF electrode contains more lattice oxygen and has better conductivity, which are partly responsible to have larger current generation as well. More importantly, this work demonstrated the excellent stability and reliability of the TiO_2_ nanotube arrays on the Ti foil, which can be considered a promising and scalable MFC bioanode for achieving a balance between low‐cost materials and high performance.

## 4 Experimental Section

*Materials and Reagents*: Titanium (Ti) foils (purity 99.99%, 0.15 mm thickness) and platinum (Pt) foils (purity 99.99%, 0.1 mm thickness) were purchased from General Research Institute for Nonferrous Metals, China. Analytical grade acetone, ethanol, propanetriol, NH_4_F, HF, K_3_[Fe(CN)_6_], CH_3_COONa, NH_4_Cl, NaCl, MgSO_4_, and HNO_3_ were purchased from Sinopharm Chemical Reagent Co., Ltd. All chemicals were used as received without further purification. Distilled water was purified using a Milli‐Q system (Millipore Filter Company, Billerica, MA).

*Fabrications of TiO_2_ Nanotube Arrays Electrode*: A piece of raw Ti foil was cut into small rectangular pieces (20 × 30 mm) which was cleaned with acetone, ethanol, and deionized water, respectively. First, it was chemically etched in HF/HNO_3_/H_2_O (1:4:5, v/v/v) mixed solution for a few seconds, and then rinsed in acetone and deionized water, respectively. Second, it was dried in air at ambient temperature (30 ± 2 °C). Anodization was performed in a two‐electrode configuration with Ti foil as the working electrode and a Pt foil served as cathode, both electrodes were placed into the HF solution (0.2 v%, 100 mL). The electrolyte was stirred at a moderate speed during the fabrication process. Anodization was performed at a constant voltage of 30 V for 30 min. The freshly prepared TiO_2_ nanotube arrays (TNA‐HF) electrode was then rinsed with distilled water and dried in a 60 °C oven overnight. The other electrolyte was composed by a mix solution of propanetriol and water with a ratio of 170:30 (v/v), and a small amount NH_4_F (0.5 wt%). After 30 min anodization, the TiO_2_ nanotube arrays (TNA‐NF) were rinsed by acetone to remove surface impurities and residual electrolyte. Along with the anodization, the surface color of Ti foil changed from purple to blue, yellow and then light red.^59^ Finally, the as‐prepared TNA‐HF and TNA‐NF foils were calcined in a muffle oven in air at 450 °C for 2 h.^40^, ^57^

*Characterization of TNA Electrode*: The morphology of the two kinds of TNA was characterized by SEM (Hitachi JSM‐7001F, Japan) with the accelerating voltage of 20 kV. The crystalline structure of the TNA was investigated by XRD technique (Thermo Fisher Scientific, USA) using Cu Kα radiation (λ = 1.5406 Å) operated at a tube current of 40 mA and a voltage of 40 kV. XRD patterns were recorded from 2θ = 20° to 80° at a scanning speed of 1° min^−1^. The XPS measurements were performed using a Thermo Fisher Scientific USA ESCA Lab250 spectrometer which consisted of monochromatic Al Kα as the X‐ray source, a hemispherical analyzer, and a sample stage with multiaxial adjustment to obtain the composition on the surface of samples.

*Electrochemical Characterization*: Electrochemical measurements were conducted using an electrochemical workstation (CHI 660C, China) in a three‐electrode system with TNA‐HF or TNA‐NF as the working electrode, a Pt foil as counter electrode, and a SCE as reference electrode.

*Reactor Construction*: All the as‐fabricated electrodes were tested in a dual‐chamber reactor, as shown in detail in Figure S6 (Supporting Information). The homemade reactor contained one anode and one cathode. The volume of the anodic chamber was equal to the cathodic chamber with 300 mL. The compartments were separated by a proton exchange membrane (PEM, Nafion 117), which was immersed in 5% H_2_O_2_, deionized water, and 0.5 mol L^−1^ H_2_SO_4_ for 2 h prior to use, respectively. The measurements were conducted at a stable ambient temperature (30 ± 2 °C). The anolyte consisted of CH_3_COONa (10 × 10^−3^ m), NH_4_Cl (5 × 10^−3^ m), NaCl (30 × 10^−3^ m), MgSO_4_ (8 × 10^−3^ m), and other trace elements.^33^ The anolyte was inoculated with the liquid from sludge storage which contains microorganism (in Figure S7, Supporting Information). The catholyte was 0.1 m K_3_[Fe(CN)_6_]. The external resistance was 1 kΩ, and the current generation data were collected using a data acquisition instrument (EM9104, China).

## Conflict of Interest

The authors declare no conflict of interest.

## Supporting information

Supplementary
