# Supplementary material for: Effects of the Structure of TiO2 Nanotube Arrays on Its Catalytic Activity for Microbial Fuel Cell
Source: Glob Chall. 2018 Oct 25;3(5):1800084. doi: 10.1002/gch2.201800084 (PMC6498118; doi:10.1002/gch2.201800084)
Supplement: Supplementary file 1 — Supplementary [file GCH2-3-1800084-s001.pdf]

Copyright WILEY-VCH Verlag GmbH & Co. KGaA, 69469 Weinheim, Germany, 2018.

Supporting Information

for *Global Challenges,* DOI: 10.1002/gch2.201800084

Effects of the Structure of TiO_2 Nanotube Arrays on Its Catalytic Activity for Microbial Fuel Cell

Tao Guo, Changzheng Wang, Ping Xu, Cuimin Feng, Shuai Si, Yajun Zhang,* Qiang Wang,* Mengtong Shi, Fengnan Yang, Jingxiao Wang, and Yang Zhang**

Copyright WILEY-VCH Verlag GmbH & Co. KGaA, 69469 Weinheim, Germany, 2016.

Supporting Information

Effects of the Structure of TiO_2 Nanotube Arrays on Its Catalytic Activity for Microbial Fuel Cell

Tao Guo,[a] Changzheng Wang,[a] Ping Xu,[a] Cuimin Feng,[a] Shuai Si,[a] Yajun Zhang,*[a] Qiang Wang,*[b] Mengtong Shi,[a] Fengnan Yang,[a] Jingxiao Wang,[a] and Yang Zhang*[c]*

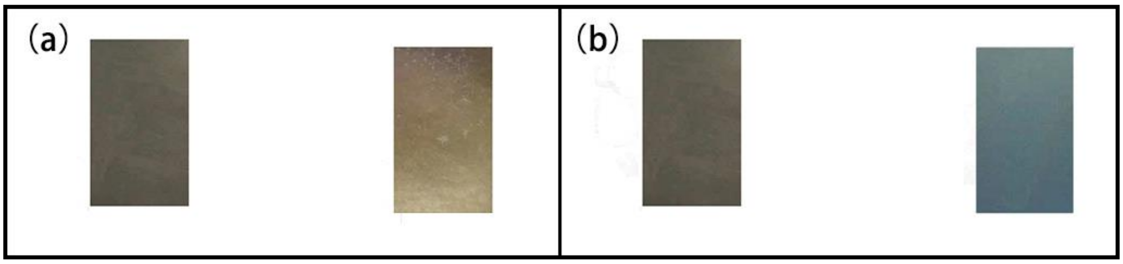

Figure S1. Morphology characterization of the TNA electrodes: (a) digital image of TNA-HF electrode before (left) and after (right) anodization. (b) digital image of TNA-NF electrode before (left) and after (right) anodization.

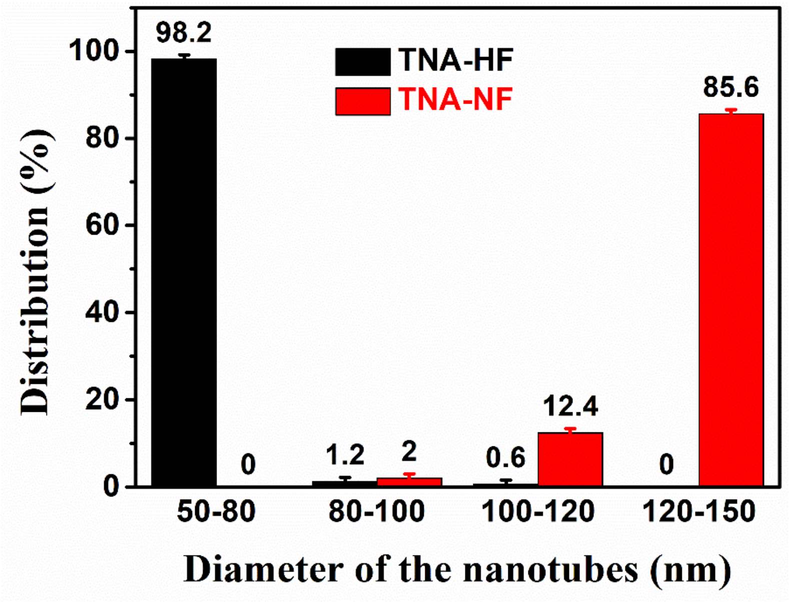

Figure S2. Nanotubes diameter distribution of the TNA-HF and TNA-NF electrodes.

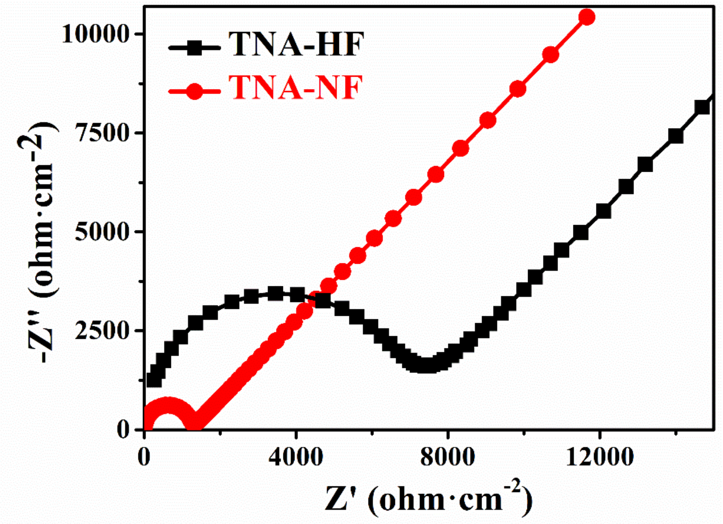

Figure S3. The electrochemical impedance of the TNA-HF and TNA-NF electrodes.

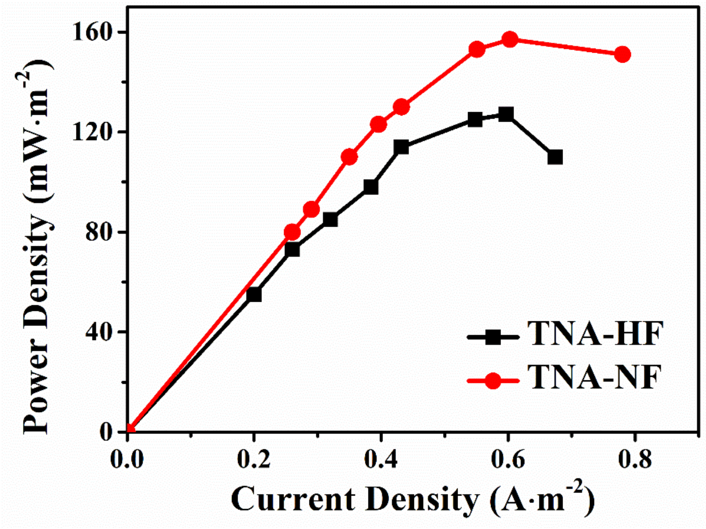

Figure S4. Power density curves of different MFC anodes.

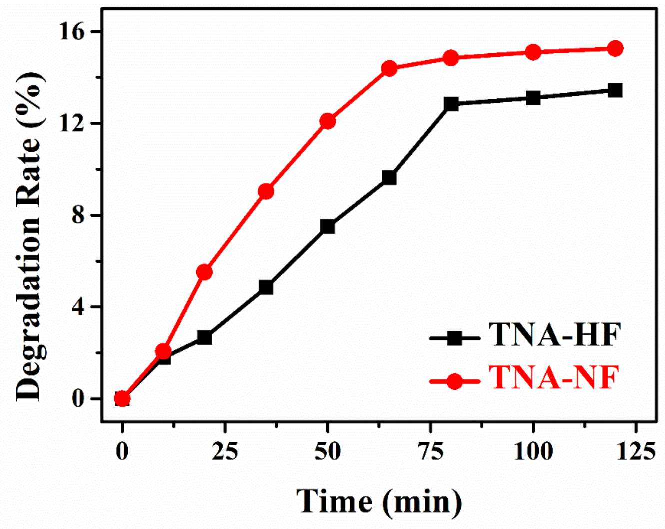

Figure S5. The degradation of MB with TNA-HF and TNA-NF under visible light.

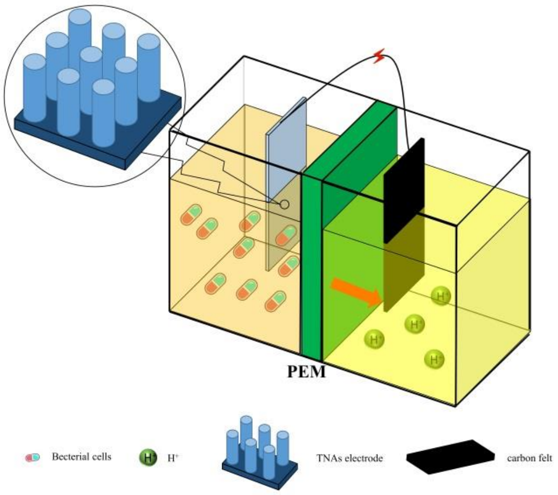

Figure S6. Schematic illustration of the MFC setup.

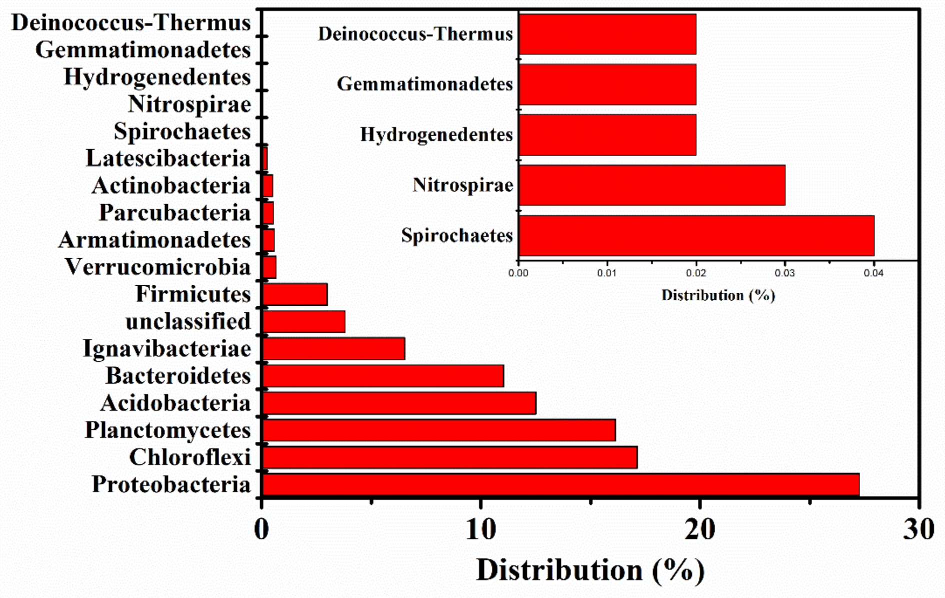

Figure S7. Distribution of micro-organisms.
